# The epidemiology of childhood intussusception in South Korea: An observational study

**DOI:** 10.1371/journal.pone.0219286

**Published:** 2019-12-20

**Authors:** Soyun Hwang, Joonghee Kim, Jae Yun Jung, Eun Mi Ham, Joong Wan Park, Hyuksool Kwon, Do Kyun Kim, Young Ho Kwak

**Affiliations:** 1 Department of Emergency Medicine, Seoul National University Hospital, Seoul, Republic of Korea; 2 Department of Emergency Medicine, Seoul National University Bundang Hospital, Seongnan-si, Gyeonggi-do, Republic of Korea; Yokohama City University, JAPAN

## Abstract

Intussusception is one of the most common causes of intestinal obstruction in young children. We report a retrospective, observational study of the epidemiology of intussusception in South Korea using the National Health Insurance Service-National Sample Cohort (NHIS-NSC). A cohort of newborns born between 2002 and 2008 was selected. The primary objective was to assess the incidence of intussusception in the pediatric population of Korea. The secondary objectives were to describe the basic epidemiological characteristics of intussusception and to identify risk factors. A total of 362 children were identified. The highest incidence of intussusception (2.6 per 1,000) was observed in children aged 1–2 years. A total of 58.8% of the children were male, and there was no significant difference in incidence according to the birth year (P = 0.804). Most of the children diagnosed with intussusception underwent air reduction, while only 0.6% had surgery. In all, 82.3% of the children were admitted to the hospital, 0.8% of them had to be admitted to the ICU, and the 6-month mortality was only 0.3%. In this retrospective, observational study, the incidence of intussusception was highest among children between 1 and 2 years of age. Most of the children underwent air reduction.

## Introduction

Intussusception is one of the most common causes of intestinal obstruction in infants and young children. It is a condition in which the proximal portion of the intestine invaginates into the more distal portion[1, 2]. The symptoms of intussusception include cyclic abdominal pain, vomiting, and bloody stools. It is usually diagnosed by radiologic means, such as ultrasonography (US). A delay in the diagnosis of intussusception can result in bowel ischemia, perforation and even death[1, 3].

Various previous papers have examined the epidemiology of intussusception. In some nationwide studies, the reported incidence of intussusception in the pediatric population was 30–60 cases per 100,000 child-years in North America, Europe, and Australasia[2, 4–7], while a higher incidence was reported in Vietnam, China, and Singapore[8–10]. After the introduction of rotavirus vaccines, other studies evaluated the risk of intussusception associated with vaccination, and these studies revealed somewhat conflicting results[3, 4, 11, 12].

We report a retrospective, observational study on the epidemiology of intussusception in South Korea. Our primary goal is to estimate the incidence of childhood intussusception in the nation and describe the epidemiology of intussusception in the pediatric population, including demographics, management, and outcomes. To date, this is the first study of intussusception using the National Health Insurance Service-National Sample Cohort.

## Methods

The institutional review board of Seoul National University Bundang Hospital approved the analysis and provided a consent waiver because the data were analyzed anonymously. (IRB X-1801-447-911)

### Data source

The data source was NHIS-NSC 2002–2013, released in 2015, which includes information on approximately 1 million people and comprises 2.2% of the total eligible population in South Korea.[13] South Korea has had a single universal health insurance coverage system for all citizens since 1989, and NHIS has been the single insurer since 2000. The total eligible population was stratified with proportional allocation according to age, sex, region, health insurance type and household income. Within each stratum, systematic stratified random sampling was conducted using the individual’s total annual medical expenses as a target variable for sampling to ensure representativeness. The NHIS-NSC contains information about the patients’ age, sex, and type of insurance; a list of diagnoses based on the International Classification of Diseases, tenth revision (ICD-10); the medical costs claimed; prescribed medications; treatments covered by the National Health Insurance; and hospital facility information. The cohort population is refreshed annually by adding representative samples of newborns each year as preexisting patients’ eligibility is disqualified by death or emigration. The institutional review board of the study hospital approved the analysis and provided a consent waiver.

### Definitions used to define newborn cohorts and primary study outcomes

A cohort of newborns for whom claim information was available from birth to the age of five years was selected (from all neonates born between 2002 and 2008). Low birth weights and preterm births were determined based on the presence of ICD codes (P07.0x or P07.1x for the former and P07.2 or P07.3x the latter). The occurrence of the primary outcome, a new event of intussusception, was defined as the new appearance of a claim record with intussusception (K56.1x) as the principal or the first additional discharge code, combined with a treatment code for air reduction (G0300, G0310, M6781 or M6782) or surgery (general anesthesia: L010x, L050x, L6x or L7x) without the same preceding event within a week prior to the event. Because the NHIS-NSC cohort does not provide the exact date of birth, we assumed that the age of each child advanced on New Year’s Day instead of on his or her birthday. For example, if a child as born in 2003, the child was defined as 0 years old in 2003 and turned 1 year old on Jan 1, 2004.

### Statistical analysis

Categorical variables were reported using frequencies and proportions, while continuous variables were reported using medians and interquartile ranges (IQRs). Wilcoxon’s rank-sum test, the chi-square test, or Fisher’s exact test was performed as appropriate for comparisons between groups.

We calculated the age- and sex-specific and age-sex standardized incidence of intussusception. We used the direct standardization method based on the population structure of the 2005 Korean census data. A seasonal difference in the number of intussusception events was tested using the Friedman test. A pairwise comparison among seasons was conducted using the pairwise Wilcoxon test with Bonferroni correction. To identify risk factors for intussusception, including any seasonal effects, we determined the presence of any intussusception event in each season for each patient’s consecutive age period from ages 1 to 5 and performed a mixed-effect logistic regression with random intercept using the seasonal observational units. The seasonal observational units at age 0 were not included in the analysis because the NHIS cohort does not provide exact birth dates.

P-values < 0.05 were considered significant. All data handling and statistical analyses were performed using the R package version 3.3.2 (R Foundation for Statistical Computing, Vienna, Austria).

## Results

A total of 63,722 newborns were identified from the cohort and included in our study (Table 1 and S1 Fig). A total of 362 (0.6%) children were diagnosed with intussusception at least once during the study period and underwent either air reduction or surgery (Table 1). Although there were slightly more boys in the total cohort population, the proportion of male children was higher in the intussusception group (N = 213, 58.8%) than in the no intussusception group (N = 32831, 51.8%; p = 0.009). Other covariates, including birth year, low birth weight, preterm birth and household income level, were not significantly different (p = 0.084, 1.000, 0.713 and 0.507, respectively). Most of the study population (63,646, 99.9%) was observed fully until the age of five without death (76, 0.1%), and there was no significant difference in mortality between the fully observed and partially observed groups (0.3% vs. 0.1%, respectively; p = 0.917). The most common age of onset was 1 year, and recurrence was rare (15/362, 4.1%).

**Table 1:** Baseline characteristics of the study cohort.

		Intussusception	No intussusception	p
		(N = 362)	(N = 63360)	
Sex				0.009
	Male	213 (58.8%)	32831 (51.8%)	
	Female	149 (41.2%)	30529 (48.2%)	
Birth year				0.804
	2002	47 (13.0%)	9499 (15.0%)	
	2003	59 (16.3%)	9360 (14.8%)	
	2004	54 (14.9%)	9246 (14.6%)	
	2005	53 (14.6%)	8480 (13.4%)	
	2006	43 (11.9%)	7807 (12.3%)	
	2007	59 (16.3%)	9653 (15.2%)	
	2008	47 (13.0%)	9315 (14.7%)	
Low birth weight		2 (0.6%)	380 (0.6%)	1.000
Preterm birth		2 (0.6%)	552 (0.9%)	0.713
Household income level				0.507
	Low	42 (11.6%)	8269 (13.1%)	
	Middle	200 (55.2%)	33185 (52.4%)	
	High	120 (33.1%)	21906 (34.6%)	
Age at first presentation				
	0 (at birth year)	54 (14.9%)		
	1	160 (44.2%)		
	2	77 (21.3%)		
	3	44 (12.2%)		
	4	20 (5.5%)		
	5	7 (1.9%)		
Number of recurrences				
	0 (no recurrence)	347 (95.9%)		
	1	11 (3.0%)		
	2	3 (0.8%)		
	3	1 (0.3%)		
Complete censoring until age five years (due to death)		1 (0.3%)	75 (0.1%)	0.917

**Fig 1:**
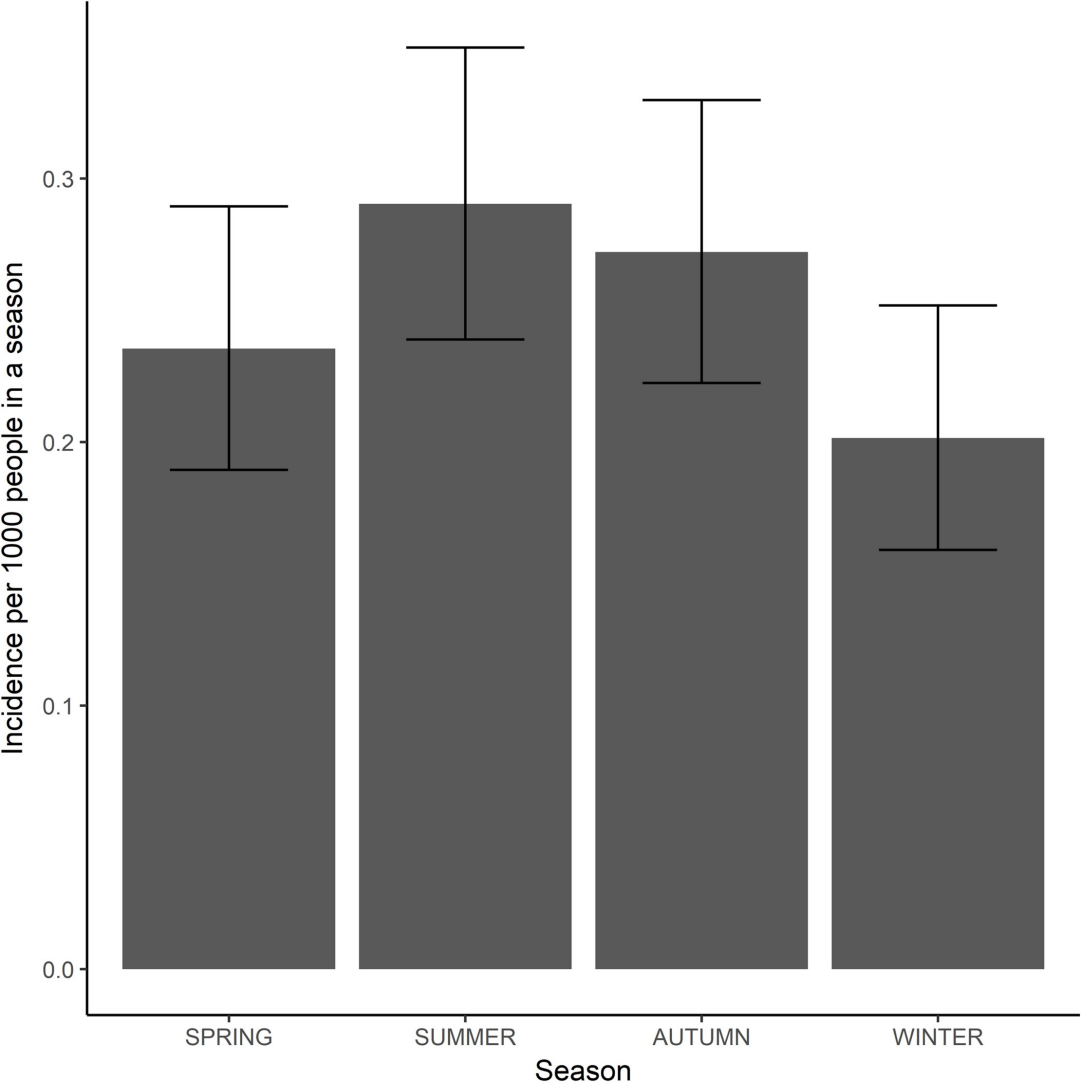
Incidence of childhood intussusception per season.

A total of 382 intussusception events were identified in the study cohort (Table 2). The most common age at occurrence was 1 year; 167 events (43.7%) occurred at that age. The most common season of occurrence was summer (n = 111, 29.1%); however, there was no significant seasonal difference between groups (p = 0.129, Fig 1). Pairwise comparisons with the Bonferroni correction also did not show any significant differences between any pairs. All intussusception cases (382/382, 100%) were treated with air reduction at least once, and two cases were treated with additional surgery (0.5%). In 315 (82.5%) cases, the patients were admitted to the hospital, and 3 (0.8%) cases were treated in the ICU. There was one case of 6-month mortality (0.3%). The primary cause of death was Burkitt lymphoma, which was first diagnosed in the outpatient setting after the patient was discharged after air reduction for intussusception. The median length of admission was 3.0 (IQR, 2–4) days, and the median cost per case was 375,240 (IQR, 261–478) USD.

**Table 2:** Characteristics of intussusception cases.

Age^a^		
	0 (at birth year)	56 (14.7%)
	1	167 (43.7%)
	2	82 (21.5%)
	3	49 (12.8%)
	4	20 (5.2%)
	5	8 (2.1%)
Season		
	Spring	90 (23.6%)
	Summer	111 (29.1%)
	Autumn	104 (27.2%)
	Winter	77 (20.2%)
Intervention		
	Air reduction	382 (100.0%)
	Surgery	2 (0.5%)
Admission		315 (82.5%)
ICU admission		3 (0.8%)
Six-month mortality		1 (0.3%)
Whole cost, KRW (IQR)		375,240 (280,270–512,140)
Length of hospital stay, days (IQR)		3 (2–4)

IQR, interquartile range

^a^. Age is 0 at birth year and increases by 1 every New Year's Day.

We calculated the age- and sex-specific incidence of intussusception (Table 3). We also calculated the age-sex standardized incidence of intussusception using the direct standardization method. The overall crude and standardized incidences from birth to age 5 were 1.09 (95% CI, 0.98–1.21) and 1.02 (95% CI, 0.92–1.12) per 1000 person-years, respectively. The incidence was highest at age 1 (2.62; 95% CI, 2.24–3.05 per 1000 person-years) in both males (2.63; 95% CI, 2.11–3.25 per 1000 person-years) and females (2.61; 95% CI, 2.07–3.24 per 1000 person-years), and the incidence decreased gradually until age 5.

**Table 3:** Age- and sex-specific incidence of intussusception.

		Male	Female	Both
Age^#1^-specific incidence				
	0 (at birth year)^#2^	1.70 (1.12–2.44)	1.82 (1.22–2.64)	1.76 (1.32–2.28)
	1	2.63 (2.11–3.25)	2.61 (2.07–3.24)	2.62 (2.24–3.05)
	2	1.51 (1.12–2.00)	1.04 (0.71–1.47)	1.29 (1.02–1.60)
	3	1.15 (0.81–1.58)	0.36 (0.18–0.64)	0.77 (0.57–1.02)
	4	0.48 (0.28–0.79)	0.13 (0.04–0.33)	0.31 (0.19–0.49)
	5	0.09 (0.02–0.27)	0.16 (0.05–0.38)	0.13 (0.05–0.25)
Crude incidence		1.22 (1.07–1.39)	0.95 (0.81–1.11)	1.09 (0.98–1.21)
Standardized incidence^#3^		1.14 (1.00–1.31)	0.88 (0.75–1.03)	1.02 (0.92–1.12)

PY, person-year; CI, confidence interval

^#1^. Age is 0 at birth year and increases by 1 every New Year's Day.

^#2^. The average follow-up duration at age 0 was assumed to be 0.5 years.

^#3^. Standardized annual incidence based on sex-age distribution in the 2005 Korean census.

We performed random intercept logistic regression using seasonal observations of new events of intussusception (Table 4). Male sex (OR, 1.36; 95% CI, 1.08–1.70; p = 0.008), younger age (OR, 20.78, 10.26, 6.13 and 2.50; 95% CI, 10.22–42.24, 4.96–21.20, 2.90–12.94 and 1.10–5.68; p-value <0.001, <0.001, <0.001 and 0.028, respectively, for ages 1, 2, 3 and 4, relative to age 5) and summer season (OR, 1.44; 95% CI, 1.05–1.97; p = 0.023 relative to winter) were identified as independent risk factors.

**Table 4:** Random intercept logistic regression model of intussusception occurrence.

		Odds ratio	*p*
Sex, male	Sex, male	1.36 (1.08–1.70)	0.008
Age ^#1^	1	20.78 (10.22–42.24)	<0.001
	2	10.26 (4.96–21.20)	<0.001
	3	6.13 (2.90–12.94)	<0.001
	4	2.50 (1.10–5.68)	0.028
	5	Reference	
Season	Spring	1.35 (0.98–1.86)	0.065
	Summer	1.44 (1.05–1.97)	0.023
	Fall	1.14 (0.82–1.58)	0.448
	Winter	Reference	
Household income level	Low	0.92 (0.65–1.30)	0.634
	Middle	Reference	
	High	0.98 (0.77–1.24)	0.842
Low birth weight	Low birth weight	1.44 (0.27–7.74)	0.673
Preterm birth	Preterm birth	0.60 (0.11–3.20)	0.547

Note: The observational unit is a seasonal observation of the occurrence of intussusception for each child.

^#1^. Age is 0 at birth year and increases by 1 every New Year's Day. Observations at age 0 were not included because seasonal observation could not be defined.

## Discussion

In this study, we assessed the epidemiological characteristics of childhood intussusception in Korea. Male children were affected more than females. The most common age at first presentation was 1 year, and recurrence was rare (4.1%). Most of the cases (82.5%) were treated in the inpatient setting with air reduction (100%), and the likelihoods of surgery, ICU admission and 6-month mortality were very low (0.5%, 0.8% and 0.3%, respectively); even the one mortality case observed was not directly caused by the condition. The incidence of childhood intussusception was 1.02 cases per 1,000 person-years in general. The highest incidence rate was observed at 1 year of age, with 2.62 cases per 1,000 person-years, and the incidence decreased gradually until age 5 years. Lastly, we identified three risk factors for childhood intussusception: male sex, younger age and the summer season. To the best of our knowledge, this is the first population-based epidemiological study of childhood intussusception in Korea.

In previous studies, the incidence of childhood intussusception varied among countries and over time[3, 14]. In one literature review, the mean incidence of intussusception worldwide was 74 per 100,000 (range: 9–328) among children <1 year of age[3]. In other studies conducted in Asia, the annual incidence of intussusception varied from 32 per 100,000 to 296 per 100,000 for infants under 1 year old[8, 10, 15, 16]. In our study, the incidence was calculated based on birth year and not exact age, so the results may not be completely comparable. Nevertheless, South Korea seems to have a relatively high incidence of intussusception compared to the average worldwide and in other countries in Asia. However, our results showed a slightly lower incidence rate compared to the previous study performed in South Korea (106 per 100,000 among children aged <5 years), although the previous study only represented one province[17].

The distribution of intussusception by age may appear different from previous studies. In most of the studies, the highest incidence was observed before 1 year of age[7, 10, 16, 18, 19]. However, in our study, in which the age group was defined based on birth year and not the exact birth month of the child, the highest incidence was observed in the 1-year-old group. This group included most of the children younger than age 1, and the actual age distribution did not differ greatly from that of previous studies.

There was no clear seasonality except that slightly fewer cases occurred in the winter and more cases occurred in the summer; in previous studies, similar results were observed regarding seasonal variation regardless of whether the country’s climate had dry/wet seasons or had four distinct seasons[10, 17]. The absence of seasonality suggests that the occurrence of intussusception is likely not related to rotavirus gastroenteritis, which is highly seasonal.

Most of the cases were treated with air reduction, a rate that was slightly higher than that observed in previous studies[7, 20]. It is known that in many developing countries, the predominant treatment modality is surgery[14, 21], and even in the same country, larger facilities tend to have a lower operative risk.[22] The high rate of air reduction suggests the high degree of proficiency with the air reduction technique in South Korea. In addition, this finding may reflect relatively easier access to hospitals in South Korea because most of the population is covered under National Health Insurance. In a previous study in the United States, children with insurance had better access to hospitals and had a reduced surgical risk[22].

Furthermore, the low mortality of childhood intussusception reflects South Korea’s relatively accessible medical system and high standards of medical care. In developed countries, the mortality rate related to intussusception is lower[14]. In addition, in previous studies, late presentation to the hospital was related to mortality[7, 16, 18], so it is possible that parents in South Korea tend to visit medical facilities early. However, there was only one case of 6-month mortality in our study, and the death was not directly related to intussusception or its complications. A review of the data revealed that this child had Burkitt lymphoma and died from the cancer.

This study has its limitations. The most notable limitation is that the children’s age was not sufficiently detailed. The cohort did not include actual birthdates, so the age grouping is inevitably inaccurate. For example, a 0-year-old group could include babies as young as 1 day old to 364 days old. Since childhood intussusception peaks before 1 year of age [7, 10, 16, 18, 19], further discrimination of age into months would have been meaningful for identifying high-risk age groups.

Additionally, this study did not consider the effect of rotavirus vaccination. In South Korea, rotavirus vaccination was introduced in 2007, but this vaccine is not covered by the National Health Insurance. As some studies consider rotavirus vaccination a potential risk factor for intussusception[3, 11, 23], analyzing and comparing intussusception rates before and after the introduction of the rotavirus vaccination in a further study would be meaningful. However, because the rotavirus vaccination is not covered by the National Health Insurance, it would be difficult to obtain information on nationwide rotavirus vaccination coverage. Second, this study did not use a complete enumeration but a sample cohort (NIHS-NSC). The actual population of South Korea is provided in the supplementary data. However, in our opinion, this national cohort sample appropriately reflects many aspects of the population structure of South Korea and has advantages over single- or multi-center studies.

## Conclusion

In this retrospective, observational study of the epidemiology of intussusception in South Korea, the incidence of intussusception was highest between the ages of 1 and 2 years. There was no significant seasonal variation, and most of the children underwent air reduction.

## Supporting information

S1 Fig Identified newborns from the cohort included in our study. (PDF)

## References

[1] Macdonald IA, Beattie TF. Intussusception presenting to a paediatric accident and emergency department. J Accid Emerg Med. 1995;12(3):182–6. Epub 1995/09/01. 10.1136/emj.12.3.182 8581242 PMC1342475

[2] Bines JE, Ivanoff B, Justice F, Mulholland K. Clinical case definition for the diagnosis of acute intussusception. J Pediatr Gastroenterol Nutr. 2004;39(5):511–8. Epub 2004/12/02. . 15572891 10.1097/00005176-200411000-00012

[3] Jiang J, Jiang B, Parashar U, Nguyen T, Bines J, Patel MM. Childhood intussusception: a literature review. PLoS One. 2013;8(7):e68482 Epub 2013/07/31. 10.1371/journal.pone.0068482 23894308 PMC3718796

[4] Justice F, Carlin J, Bines J. Changing epidemiology of intussusception in Australia. J Paediatr Child Health. 2005;41(9–10):475–8. 10.1111/j.1440-1754.2005.00686.x . 16150062

[5] Palupi-Baroto R, Lee KJ, Carlin JB, Bines JE. Intussusception in Australia: epidemiology prior to the introduction of rotavirus vaccine. Aust N Z J Public Health. 2015;39(1):11–4. 10.1111/1753-6405.12297 . 25558780

[6] Rosie B, Dalziel S, Wilson E, Best EJ. Epidemiology of intussusception in New Zealand pre-rotavirus vaccination. N Z Med J. 2016;129(1442):36–45. . 27657157

[7] Jenke AC, Klaassen-Mielke R, Zilbauer M, Heininger U, Trampisch H, Wirth S. Intussusception: incidence and treatment-insights from the nationwide German surveillance. J Pediatr Gastroenterol Nutr. 2011;52(4):446–51. Epub 2011/03/19. 10.1097/MPG.0b013e31820e1bec . 21415671

[8] Van Trang N, Le Nguyen NT, Dao HT, Ho VL, Tran DT, Loewen J, et al Incidence and Epidemiology of Intussusception among Infants in Ho Chi Minh City, Vietnam. J Pediatr. 2014;164(2):366–71. 10.1016/j.jpeds.2013.10.006 . 24238857

[9] Cui P, Liu N, Li J, Huang T, Ge H, Wu Q, et al [Epidemiology of intussusception related hospitalizations in children aged <2 years in Suzhou, 2007–2013]. Zhonghua Liu Xing Bing Xue Za Zhi. 2016;37(3):410–4. 10.3760/cma.j.issn.0254-6450.2016.03.025 . 27005548

[10] Boudville IC, Phua KB, Quak SH, Lee BW, Han HH, Verstraeten T, et al The epidemiology of paediatric intussusception in Singapore: 1997 to 2004. Ann Acad Med Singapore. 2006;35(10):674–9. . 17102889

[11] Haber P, Patel M, Pan Y, Baggs J, Haber M, Museru O, et al Intussusception after rotavirus vaccines reported to US VAERS, 2006–2012. Pediatrics. 2013;131(6):1042–9. Epub 2013/05/15. 10.1542/peds.2012-2554 . 23669521

[12] Kim KY, Kim DS. Relationship between Pentavalent Rotavirus Vaccine and Intussusception: A Retrospective Study at a Single Center in Korea. Yonsei Med J. 2017;58(3):631–6. Epub 2017/03/24. 10.3349/ymj.2017.58.3.631 28332371 PMC5368151

[13] Lee J, Lee JS, Park SH, Shin SA, Kim K. Cohort Profile: The National Health Insurance Service-National Sample Cohort (NHIS-NSC), South Korea. Int J Epidemiol. 2017;46(2):e15 Epub 2016/01/30. 10.1093/ije/dyv319 . 26822938

[14] Bines J, Ivanoff B. Acute Intussusception in Infants and Children: Incidence, Clinical Presentation and Management: A Global Perspective. Geneva, Switzerland: World Health Organization. 2002;Report 02.19.

[15] Noguchi A, Nakagomi T, Kimura S, Takahashi Y, Matsuno K, Koizumi H, et al Incidence of intussusception as studied from a hospital-based retrospective survey over a 10-year period (2001–2010) in Akita Prefecture, Japan. Jpn J Infect Dis. 2012;65(4):301–5. Epub 2012/07/21. . 22814151

[16] Takeuchi M, Osamura T, Yasunaga H, Horiguchi H, Hashimoto H, Matsuda S. Intussusception among Japanese children: an epidemiologic study using an administrative database. BMC Pediatr. 2012;12:36 Epub 2012/03/24. 10.1186/1471-2431-12-36 22439793 PMC3350444

[17] Jo DS, Nyambat B, Kim JS, Jang YT, Ng TL, Bock HL, et al Population-based incidence and burden of childhood intussusception in Jeonbuk Province, South Korea. Int J Infect Dis. 2009;13(6):e383–8. Epub 2009/04/14. 10.1016/j.ijid.2009.01.011 . 19362503

[18] Satter SM, Aliabadi N, Yen C, Gastanaduy PA, Ahmed M, Mamun A, et al Epidemiology of childhood intussusception in Bangladesh: Findings from an active national hospital based surveillance system, 2012–2016. Vaccine. 2017 Epub 2017/09/25. 10.1016/j.vaccine.2017.08.092 . 28941622 PMC5864564

[19] Mehendale S, Kumar CP, Venkatasubramanian S, Prasanna T. Intussusception in Children Aged Less than Five years. Indian J Pediatr. 2016;83(10):1087–92. 10.1007/s12098-016-2152-9 . 27211600

[20] Blanch AJ, Perel SB, Acworth JP. Paediatric intussusception: epidemiology and outcome. Emerg Med Australas. 2007;19(1):45–50. 10.1111/j.1742-6723.2007.00923.x . 17305660

[21] Gadisa A, Tadesse A, Hailemariam B. Patterns and Seasonal Variation of Intussusception in Children: A Retrospective Analysis of Cases Operated in a Tertiary Hospital in Ethiopia. Ethiop Med J. 2016;54(1):9–15. . 27191025

[22] Bratton SL, Haberkern CM, Waldhausen JH, Sawin RS, Allison JW. Intussusception: hospital size and risk of surgery. Pediatrics. 2001;107(2):299–303. Epub 2001/02/07. 10.1542/peds.107.2.299 . 11158462

[23] Desai R, Cortese MM, Meltzer MI, Shankar M, Tate JE, Yen C, et al Potential intussusception risk versus benefits of rotavirus vaccination in the United States. Pediatr Infect Dis J. 2013;32(1):1–7. Epub 2012/08/30. 10.1097/INF.0b013e318270362c . 22929172 PMC5714269

