# Supplementary material for: The epidemiology of childhood intussusception in South Korea: An observational study
Source: PLoS One. 2019 Dec 20;14(12):e0219286. doi: 10.1371/journal.pone.0219286 (PMC6924652; doi:10.1371/journal.pone.0219286)
Supplement: S1 Fig — (PDF) [file pone.0219286.s001.pdf]

Birth year	2002	2003	2004	2005	2006	2007	2008	2009	2010	2011	2012	2013	Number of neonates by birth year (N=63,722)
Birth age by birth year	0	1	2	3	4	5							9,546
		0	1	2	3	4	5						9,419
			0	1	2	3	4	5					9,300
				0	1	2	3	4	5				8,533
					0	1	2	3	4	5			7,850
						0	1	2	3	4	5		9,712
							0	1	2	3	4	5	9,362
